# Functional brain connectivity from the fetal period through infancy: A narrative review of resting-state fMRI studies

**DOI:** 10.1016/j.dcn.2026.101804

**Published:** 2026-08-29

**Authors:** Xin Tang, Fangwei Yuan, Xinyu Lin, Chang Liu, Rong Ju

**Affiliations:** a Chengdu Women's and Children's Central Hospital, School of Medicine, University of Electronic Science and Technology of China, Chengdu 611731, China; b The Key Laboratory for Computer Systems of State Ethnic Affairs Commission, Southwest Minzu University, Chengdu, Sichuan 610225, China

**Keywords:** Early brain development, Resting-state functional magnetic resonance imaging, Functional connectivity, Prematurity, Early-life environment, Neurodevelopmental outcomes

## Abstract

The interval from the fetal period through infancy is critical for the formation and reorganization of large-scale functional brain networks. Resting-state functional magnetic resonance imaging (rs-fMRI) requires no task performance and provides a means of characterizing whole-brain functional organization during this interval. This narrative review synthesizes human rs-fMRI evidence on typical functional network development, prematurity-related differences, associations with maternal and early caregiving environments, and links between early functional connectivity and subsequent neurodevelopmental outcomes. Overall, early functional network development shows a degree of hierarchical organization: primary sensorimotor systems show relatively stable organization earlier, whereas higher-order association systems undergo more prolonged refinement. Local specialization and cross-network integration overlap, with patterns varying by connection distance, brain region, and individual. We further examine associations of prematurity, maternal psychological and social environments, metabolic and immune status, substance and environmental chemical exposures, neonatal intensive care unit (NICU) experiences, and early sensory and social interactions with functional network differences. We also summarize prospective evidence relating fetal, neonatal, and infant connectivity features to later motor, language, cognitive, and socioemotional outcomes, as well as autism spectrum disorder-related risk measures. These findings suggest that early functional connectivity may contain information about developmental vulnerability before behavioral or clinical features become apparent. Finally, we discuss methodological challenges related to scan state (sleep or wakefulness), head motion, brain parcellation, and analytical pipelines, and consider how multisite longitudinal studies, standardized data acquisition, and independent-cohort validation could improve reproducibility and support future clinical translation.

## Highlights

• This review integrates rs-fMRI evidence from the fetal period through infancy.
• Primary networks organize earlier; association systems show prolonged refinement.
• Prematurity and early-life environments are associated with connectivity differences.
• Early connectivity is prospectively associated with later developmental outcomes.
• Sleep state, motion, physiology, and analysis choices shape connectivity estimates.

## 1 Introduction

From fetal life through infancy, the human brain undergoes rapid and interdependent structural and functional reorganization (H. Chen et al., 2021; Estrin and Bhavnani, 2020). Neuronal migration, cortical folding, synaptogenesis, dendritic development, glial proliferation, and myelination contribute to brain growth and neural circuit formation (Akula et al., 2023, Semple et al., 2013, Tau and Peterson, 2010). Spontaneous neural activity is present during early brain development, and human fetal studies have detected functional coupling between homotopic regions and the emergence of large-scale functional networks (Desrosiers et al., 2024, Luhmann et al., 2016, Turk et al., 2019). These early structural and functional changes provide a foundation for later sensory, motor, cognitive, and socioemotional abilities, but rapid development also entails heightened environmental sensitivity. Maternal psychological and immunometabolic conditions, medication or substance exposures, prematurity, systemic illness, and adverse early caregiving contexts have each been investigated in relation to early brain-network organization (Jia et al., 2023, Thomason, 2020, Thomason et al., 2021a, Tooley et al., 2021). Characterizing typical functional network development and its possible deviations is therefore important for understanding neurodevelopmental vulnerability and identifying potential windows for early intervention.

Several non-invasive methods have been used to study fetal and infant brain function, including electroencephalography (EEG), magnetoencephalography (MEG), functional near-infrared spectroscopy (fNIRS), and functional magnetic resonance imaging (fMRI). EEG records electrical potentials generated by neuronal activity, whereas MEG detects the associated magnetic fields; both provide high temporal resolution. Before fetal EEG signals reach the maternal body surface, they are attenuated by vernix caseosa and maternal tissues and are further affected by fetal posture and electrode placement. MEG is generally less affected by differences in tissue conductivity, but its routine use is limited by the low amplitude of fetal neuromagnetic signals, fetal movement and changes in posture, source-localization difficulties, magnetic-shielding requirements, and limited equipment availability (Vasung et al., 2019). fNIRS is portable and suitable for measurements in awake infants, but it primarily reflects hemodynamic changes in superficial cortical tissue and has limited spatial coverage and access to deep structures (Pinti et al., 2020). Resting-state functional magnetic resonance imaging (rs-fMRI) does not require participants to perform a task and can be acquired during fetal life or during natural sleep or quiet wakefulness in infants, making it particularly suitable for early developmental research. The BOLD contrast is sensitive to changes in blood oxygenation (Ogawa et al., 1990), and rs-fMRI functional connectivity is commonly operationalized as temporal correlations between spontaneous low-frequency BOLD fluctuations in different brain regions (Elam et al., 2021). Importantly, BOLD functional connectivity is not equivalent to direct neuronal synchrony or structural connectivity, and its estimation may also be affected by sleep state, neurovascular coupling, respiration, head motion, and analytic procedures.

Several recent reviews have synthesized early brain connectome research from complementary perspectives. Desrosiers et al. (2024) systematically reviewed rs-fMRI evidence from the prenatal and neonatal stages, emphasizing developmental patterns of functional connectivity and methodological limitations. Arichi (2024) discussed the formation of large-scale human neural circuits from a multimodal in vivo imaging perspective. Gao (2025) proposed a hierarchical account in which early functional networks become progressively integrated from primary systems toward higher-order cognitive and socioemotional systems. These reviews provide important reference points, while differing in temporal scope, imaging modalities, and central questions.

Building on this work, the present review uses human rs-fMRI evidence as its central focus, with the fetal period through the first postnatal year as the core developmental window; studies in toddlerhood and longer-term follow-up are included where necessary to interpret developmental patterns and brain-behavior relationships. We first summarize the emergence, topological organization, distance dependence, and hub reconfiguration of functional networks during typical development. We then examine network differences associated with prematurity and other high-risk conditions. Next, we consider early environmental associations by grouping evidence on maternal psychological and social contexts, metabolic and immune status, medication and substance exposure, sensory experience, social interaction, and neonatal intensive care unit (NICU) care. We subsequently evaluate prospective associations between early connectivity and cognitive, language, motor, and socioemotional outcomes, before addressing methodological challenges related to sleep state, head motion, brain parcellation, and analytic pipelines.

This article is a narrative review. The literature was selected purposively to represent typical resting-state functional connectivity development from fetal life through infancy, prematurity-related differences, perinatal environmental factors, brain-behavior associations, and major methodological challenges. Priority was given to human rs-fMRI primary studies directly relevant to these topics, with particular consideration of sample size, study design, longitudinal follow-up, behavioral outcomes, and level of validation. To support the physiological and methodological interpretation of functional connectivity, we also drew selectively on relevant studies using other non-invasive methods, including EEG and fNIRS, as well as directly pertinent methodological literature. Table 1, Table 2 summarize representative rather than exhaustive studies.

**Table 1:** Representative prenatal and early postnatal exposures, clinical experiences, and interventions associated with early functional connectivity differences.

*Representative studies; illustrative rather than exhaustive*					
**Exposure, clinical experience, or intervention**	**Representative study/studies**	**Imaging sample and design**	**Developmental stage at rs-fMRI**	**Reported functional connectivity findings**	**Follow-up evidence and interpretive limits**
Maternal prenatal stress trajectories	Marr et al. (2023)	Neonatal imaging subsample n = 60; longitudinal behavioral cohort N = 115; independent behavioral replication cohort N = 2156	Neonatal period, shortly after birth	Increasing or late-pregnancy peak stress trajectories were associated with stronger neonatal amygdala-vmPFC and/or amygdala-anterior insula connectivity.	Stress trajectory-negative affect associations were replicated behaviorally, but the imaging result was not independently replicated. The imaging and mediation analyses were exploratory and underpowered.
Maternal childhood adversity	Hendrix et al. (2021); Van Den Heuvel et al. (2023)	n = 48 Black mother-neonate dyads; N = 89 mother-fetus dyads; observational cohorts using retrospective maternal reports	Neonatal scan at approximately 1 month; fetal scan at mean 32.6 weeks' gestation (range approximately 21–40 weeks)	Maternal emotional neglect was associated with stronger neonatal amygdala-vmPFC and amygdala-dACC connectivity. Maternal childhood maltreatment was associated with higher fetal amygdala connectivity to left prefrontal/premotor regions and lower connectivity to right premotor and brainstem regions.	No later behavioral validation was reported. Current findings do not establish an intergenerational causal mechanism and may be influenced by current maternal psychological and social factors.
Prenatal socioeconomic and social disadvantage	Ramphal et al. (2020); Nielsen et al. (2024)	Neonatal imaging n = 112 with behavioral follow-up n = 46; discovery imaging n = 261 with an independent validation sample n = 151	Birth or term-equivalent age; first weeks after birth	Lower socioeconomic status was associated with striatal and ventrolateral prefrontal connectivity differences. Multivariate neonatal connectivity patterns were associated with prenatal social disadvantage across association, attention, somatomotor, and amygdala-related systems.	Selected frontostriatal connections statistically mediated socioeconomic status-behavior associations in a small follow-up subsample. The social disadvantage-connectivity mapping was externally validated, but causal biological embedding was not tested.
Prenatal neighborhood crime exposure	Brady et al. (2022)	n = 319 neonatal scans from a prenatal cohort of N = 399; observational geocoded exposure study	Neonatal period	Higher property and violent crime exposure was associated with weaker thalamus-anterior DMN connectivity. Violent crime was also associated with weaker amygdala-hippocampus connectivity.	Maternal psychosocial stress partially mediated the thalamus-anterior DMN associations. Crime estimates were area-level exposures, and the study does not establish a causal effect on neonatal networks.
Maternal experiences of discrimination or racism	Spann et al. (2024); Kral et al. (2024)	Imaging n = 38 and n = 25; observational studies using maternal self-report	Within 6 weeks after birth; approximately 2 weeks after birth	Discrimination was associated with weaker amygdala-prefrontal and stronger amygdala-fusiform connectivity. Racism experiences were associated with stronger amygdala-visual cortex/thalamus and hippocampal visual/temporoparietal connectivity.	Both studies were small and lacked later behavioral validation. Hypervigilance was proposed as a possible interpretation but was not directly measured.
Maternal prepregnancy adiposity	Salzwedel et al. (2019); Spann et al. (2020); Rajasilta et al. (2021)	n = 38 (23 normal-BMI, 15 high-BMI); imaging n = 45; n = 21; observational cohorts	Neonatal period, generally within the first weeks after birth	Higher maternal BMI was associated with differences across sensory, reward, control, and motor connections; greater thalamic global/local connectivity with lower frontothalamic connectivity; and altered left superior frontal ReHo and distal connectivity.	Samples were small and no later behavioral validation was reported. Fetal estimated weight did not significantly mediate the thalamic connectivity associations in Spann et al. (2020).
Maternal systemic inflammation	Spann et al. (2018); Rudolph et al. (2018); Graham et al. (2018)	Immune data n = 36, Bayley follow-up n = 21; N = 84 with working-memory follow-up n = 46; functional MRI n = 70 from N = 86	Neonatal period, shortly after birth	Maternal IL-6 and/or CRP was associated with neonatal salience-network, large-scale, and amygdala connectivity differences. Internal cross-validation estimated maternal IL-6 from neonatal connectivity in Rudolph et al. (2018).	Later cognition, working memory, or impulse control was associated with maternal inflammation. Amygdala measures statistically mediated the IL-6-impulse control association in Graham et al. (2018), whereas neonatal connectivity did not mediate the inflammation-cognition association in Spann et al. (2018). No external imaging validation was reported.
Prenatal PM2.5 exposure	Blanco-Hinojo et al. (2026)	n = 61 neonates; observational exposure study	Mean postnatal age approximately 29 days	Higher prenatal PM2.5 exposure was associated with greater local iso-distant average correlation, mainly in sensorimotor regions, with distance-dependent effects in premotor and supplementary motor cortices.	The findings could reflect delayed segregation of primary systems or accelerated local organization within the sensorimotor hierarchy. No later behavioral follow-up was available.
Antenatal magnesium sulfate treatment	Ufkes et al. (2024)	n = 45 preterm infants nested within a randomized placebo-controlled trial: 24 exposed to MgSO4 and 21 placebo	Term-equivalent age	MgSO4 exposure was associated with greater voxelwise connectivity in temporal, occipital, and deep-gray regions and with differences in selected graph metrics.	The imaging sample was small, and connectivity was not directly linked to neurodevelopmental outcome in this study. The findings should not be labeled a demonstrated neuroprotective imaging effect.
Prenatal opioid exposure	Radhakrishnan et al. (2022a)	19 opioid-exposed infants and 20 controls; observational case-control study	Neonatal or early postnatal period	Prenatal opioid exposure was associated with thalamocortical connectivity differences, and selected connections correlated with modified Finnegan scores indexing neonatal opioid withdrawal severity.	Maternal polysubstance use, smoking, and psychological conditions modified some associations. The study did not provide later neurodevelopmental validation.
Skin-breaking procedures during NICU care	Cook et al. (2023)	148 very/extremely preterm infants and 99 term controls; imaging-behavior follow-up subsample approximately n = 41	Term-equivalent age; outcomes at 18 months	More skin-breaking procedures were associated with stronger intra-cerebellar and cerebellar-limbic/paralimbic connectivity. Procedure counts were also associated with autism-risk, motor, and language scores.	Only selected connectivity-language associations survived correction; connectivity was not uniformly related to all outcomes. Illness severity and co-occurring NICU care exposures limit causal interpretation.
NICU music-based intervention	Lordier et al. (2019b); Haslbeck et al. (2020), (2021), (2023)	Lordier final rs-fMRI groups: 14 music-exposed preterm, 15 preterm controls, 16 term controls; Haslbeck randomized 82 infants, final MRI n = 40 and fMRI n = 34	Preterm infants scanned at term-equivalent age	Music exposure was associated with stronger coupling among auditory, sensorimotor, salience, superior frontal, thalamic, and posterior cingulate/precuneus systems. Creative music therapy was associated with lower thalamocortical lag and selected integration differences.	Both imaging analyses had substantial attrition and small final samples. A randomized 2-year follow-up found no significant group benefit. A separate small nonrandomized feasibility follow-up found no 2-year difference and only a 5-year cognitive trend. Durable benefit is not established.

Note. Studies were selected to represent the principal exposure and intervention domains discussed in Section 4; the table is illustrative rather than exhaustive. Sample sizes refer to the imaging sample unless otherwise stated.

Abbreviations: BMI, body mass index; CRP, C-reactive protein; dACC, dorsal anterior cingulate cortex; DMN, default mode network; IL-6, interleukin-6; MgSO4, magnesium sulfate; NICU, neonatal intensive care unit; PM2.5, particulate matter with aerodynamic diameter < =2.5 micrometers; ReHo, regional homogeneity; rs-fMRI, resting-state functional magnetic resonance imaging; vmPFC, ventromedial prefrontal cortex.

**Table 2:** Representative early-life functional connectivity features and their prospective associations with later neurodevelopmental outcomes.

Representative studies; illustrative rather than exhaustive					
**Developmental or clinical domain**	**Representative study**	**Early imaging sample and age**	**Early functional connectivity feature**	**Later outcome and assessment age**	**Evidence level and main limitation**
Autism-related traits from the fetal period	Chen et al. (2026)	N = 62 fetal scans; mean gestational age 31.3 weeks (range 24.4–38.0 weeks)	Cingulate-left temporal and right prefrontal-left opercular network-pair connectivity was associated with later BITSEA autism-related trait scores.	Higher parent-reported continuous autism-related traits at age 3 years	Prospective observational association. The outcome was a continuous parent-report trait measure, not an autism diagnosis; no external validation was performed.
Autism-related social-communication vulnerability	Scheinost et al. (2022b)	n = 53 neonates: 12 with high familial likelihood and 41 with low likelihood; mean 44.0 weeks PMA; FYI follow-up n = 25	Lower left anterior insula-left amygdala connectivity was associated with higher later FYI social-communication risk scores.	FYI assessment at 12–18 months (mean 17.3 months)	Prospective observational association. The high-likelihood group and behavioral follow-up sample were small; FYI scores are not a clinical autism diagnosis and the finding lacks external validation.
Outcome after neonatal acute brain injury	Boerwinkle et al., 2022, Boerwinkle et al., 2024	Same retrospective clinical cohort, baseline N = 40; rs-fMRI acquired during hospitalization within 31 days of suspected brain injury	Abnormalities in basal ganglia, default mode, language/frontoparietal, and resting-state seizure-onset-zone networks showed domain-specific associations with later outcomes.	Mortality, PCPC, developmental and motor outcomes at approximately 6 months and median 30.5 months; later epilepsy	Longitudinal associations in the same clinical cohort, not independent replication. Deaths and loss to follow-up reduced the later sample; no externally validated prognostic threshold was established.
Language and reading	Tang et al. (2024)	76 infants scanned at mean 9.3 + /- 3.5 months; phonological follow-up n = 61; reading follow-up n = 41	Stronger connectivity within an inferior frontal gyrus module of the infant language network was prospectively associated with later language-related skills.	Phonological skills at mean 5.8 years and word reading at mean 8.1 years	Prospective association with statistical mediation by phonological skill. Follow-up samples declined over time, and the pathway was not tested in an independent cohort.
Cognitive and motor outcomes after very preterm birth	Toulmin et al. (2021)	n = 102 very preterm infants without major focal lesions; rs-fMRI at term-equivalent age	Thalamus-premotor association cortex connectivity was associated with motor scores, whereas thalamus-primary sensorimotor connectivity was associated with cognitive scores.	Standardized cognitive and motor assessment at 20 months	Prospective observational association with family-wise error correction. No external cohort validation or clinical decision threshold was evaluated.
Motor outcome after high-grade brain injury in very preterm infants	Cyr et al. (2022)	n = 107 very preterm infants: 55 with high-grade injury and 52 without; 87 had 2-year follow-up; scan at 35–45 weeks PMA	Stronger interhemispheric motor cortex connectivity was associated with better gross motor outcome only in the brain-injured subgroup; the connectivity-by-injury interaction was significant.	Gross motor outcome at 2 years corrected age	Prospective subgroup-specific association. A compensatory mechanism was not directly tested, and no external validation was performed.
Childhood emotion moderation after very preterm birth	Kanel et al. (2022)	n = 129 very preterm infants; scan at mean 42.2 weeks PMA	Neonatal amygdala connectivity with parahippocampal, occipital, and thalamic regions was associated with later Emotion Moderation scores.	Composite Emotion Moderation measure at age 4–7 years (mean 5.0 years)	Prospective observational association. No significant associations were found for Social Function or Empathy, and no external validation was performed.
Negative affect and internalizing symptoms	Ravi et al. (2023)	n = 72 infants with usable 1-month rs-fMRI; outcome-specific analytic samples varied, including n = 20 for 18-month internalizing symptoms	Stronger within-network DMN connectivity at approximately 1 month was associated with lower negative affect or crying and fewer later internalizing symptoms.	Negative affect or crying at 1 and 6 months; internalizing symptoms at 18 months	Prospective observational associations. The later analytic samples were very small and the findings require independent replication.
Callous-unemotional traits, empathy, and prosociality	Brady et al. (2024a)	N = 283 newborns; mean 41.2 weeks PMA (range 37–45 weeks)	Stronger cingulo-opercular network-medial prefrontal cortex connectivity was associated with later behavioral traits.	Higher callous-unemotional traits at age 3 and lower empathy/prosociality across ages 1–3 years	Prospective observational association that remained after adjustment for parent traits and child externalizing symptoms. Outcomes were parent-reported and the connectivity finding was not externally validated.
Fearful temperament	Filippi et al. (2025)	180 infants contributing 396 scans from birth to 28 months; age-2 fear data n = 93	DAN-FPN and DMN-SN connectivity decreased with age. A smaller DAN-FPN decrease and selected initial DAN-FPN/DAN-SN levels were associated with greater or increasing fearfulness.	Parent-reported fearfulness at 20–27 months and longitudinal fear trajectories	Repeated-measures longitudinal association. Fear data were incomplete, causal direction was not established, and there was no independent external validation.

Note. The table summarizes representative longitudinal evidence rather than validated biomarkers.

Abbreviations: BITSEA, Brief Infant-Toddler Social and Emotional Assessment; DAN, dorsal attention network; DMN, default mode network; FPN, frontoparietal network; FYI, First Year Inventory; PCPC, Pediatric Cerebral Performance Category; PMA, postmenstrual age; rs-fMRI, resting-state functional magnetic resonance imaging; SN, salience network.

## 2 Normative functional network development

Available rs-fMRI evidence suggests that functional network development from the fetal period through infancy follows a broadly hierarchical, but not strictly sequential, pattern. Primary sensorimotor systems generally acquire relatively stable spatial organization earlier, whereas higher-order association networks and inter-network coordination undergo more prolonged refinement. Functional coupling between subcortical structures, particularly the thalamus, and cortical networks is also reorganized during this period. Local specialization, modular differentiation, and distributed integration proceed concurrently, with age-related patterns varying according to connection distance, brain region, and analytical measure. Network modularity, global and local efficiency, and hub distribution likewise change across development. Because most fetal studies have used cross-sectional or mixed designs, these findings are best interpreted as population-level developmental trends rather than a fixed sequence followed by every individual.

### 2.1 From the fetal period to the neonatal period

Wen et al. (2022) found that the intrinsic connectivity pattern linking regions involved in tool processing in the adult human brain was already detectable in neonates, whereas a comparable organization was not observed among homologous regions in the macaque sample. This early connectivity pattern may therefore provide an initial neural scaffold for the later development of complex tool-related abilities. Consistent with this view, several neonatal rs-fMRI studies have shown that, before the corresponding behavioral capacities are fully expressed and while postnatal experience remains relatively limited, some higher-order association cortices already exhibit intrinsic connectivity patterns that partially resemble their adult functional network counterparts (Barttfeld et al., 2018, Kamps et al., 2020, Li et al., 2020, Zhu et al., 2025). These findings do not imply that higher-order cognitive functions are mature at birth. Rather, they suggest that early connectivity biases may provide an initial organizational scaffold that is subsequently refined and reorganized through maturation and experience.

#### 2.1.1 Topological reorganization and the segregation-integration balance

By mid-to-late gestation, the fetal functional connectome already exhibits small-world topology, modular organization, and rich-club organization (De Asis-Cruz et al., 2021, Nazari and Salehi, 2023, Turk et al., 2019). With increasing gestational age, small-worldness, local efficiency, and modularity tend to decline (De Asis-Cruz et al., 2021), possibly indicating continued adjustment in the balance between network segregation and integration during the perinatal period. Hub nodes are considered core brain regions that play key topological roles in information integration within brain networks and across modules. During fetal life, major hubs first emerge in primary sensorimotor regions, such as the visual and motor cortices, and in structures such as the cerebellum; however, some higher-order association regions, including the inferior temporal gyrus and angular gyrus, already show hub-like features (Van Den Heuvel et al., 2018). These hubs are more prominently distributed in the left hemisphere, suggesting that functional lateralization may begin to emerge in utero (Van Den Heuvel et al., 2018). Turk et al. (2019) further found that hubs were mainly located in the temporal lobe, midline frontal regions, and insular cortex, while also involving primary somatosensory and some visual association areas. During the perinatal transition from 25 to 55 weeks, one study showed that the subcortical network, particularly the thalamus, appeared to be the only network with a significant increase in local efficiency around birth (Ji et al., 2024). This may suggest a central role for subcortical circuits in perinatal neural adaptation and in the transition from the intrauterine to the extrauterine environment. Overall, by term-equivalent age, primary networks have established a topology broadly comparable to that of the adult brain, whereas higher-order association networks remain under ongoing organization (Eyre et al., 2021, Truzzi and Cusack, 2023). This pattern is consistent with a general developmental progression from primary systems toward higher-order association systems. However, except for the few studies that include repeated measurements, most fetal evidence still comes from cross-sectional analyses, and hub localization and graph-theoretical results depend on the atlas, thresholding strategy, and definition of centrality used. Therefore, the sequence described above should not be interpreted as an established fixed within-individual developmental trajectory.

#### 2.1.2 Subcortical-cortical coupling and functional lateralization

Early functional network development involves both subcortical-cortical and cortico-cortical coupling. In a seed-based study of 16 fetuses scanned between 25 and 32 weeks of gestation, the thalamus, striatum, hippocampus, amygdala, and other subcortical structures showed functional coupling with multiple cortical territories (Canini et al., 2020). In the mature brain, these territories contribute to sensorimotor processing, salience-related functions, and memory and learning. Their detection in fetuses should therefore be interpreted as evidence of emerging coupling, rather than evidence that the corresponding cognitive functions are already operational.

This functional organization develops against a well-characterized structural background. Thalamocortical axonal outgrowth begins during the first trimester, followed by a period of accumulation within the subplate and subsequent ingrowth into the cortical plate at approximately 23–24 gestational weeks. These pathways provide important input to the developing cortex and contribute to cortical areal differentiation and the establishment of circuits supporting sensory integration (Arichi, 2024). Consistent with this developmental framework, Taymourtash et al. (2023) found age-related strengthening of multiple thalamocortical and cortico-cortical connections in fetuses studied between 19 and 40 weeks of gestation, with the most rapid thalamocortical increase occurring around 29–31 weeks. Homotopic cortical regions initially showed broadly mirrored connectivity profiles, but the similarity between these profiles decreased with gestational age, suggesting increasing differentiation of cortical interactions.

Localized functional asymmetry is also detectable before birth. Taymourtash et al. (2023) reported left-lateralized connectivity in superior, medial, and inferior temporal regions overlapping territories later associated with receptive language processing, although an age-related increase in laterality was statistically evident only in the inferior temporal region. By the neonatal period, asymmetry is more clearly distributed across several functional systems. Auditory and temporoparietal networks show localized leftward asymmetry, whereas dorsal and ventral visual association networks show predominantly rightward asymmetry (Williams et al., 2023).

#### 2.1.3 Rudimentary organization of higher-order networks and differentiation of inter-network interactions

**Fig. 1:**
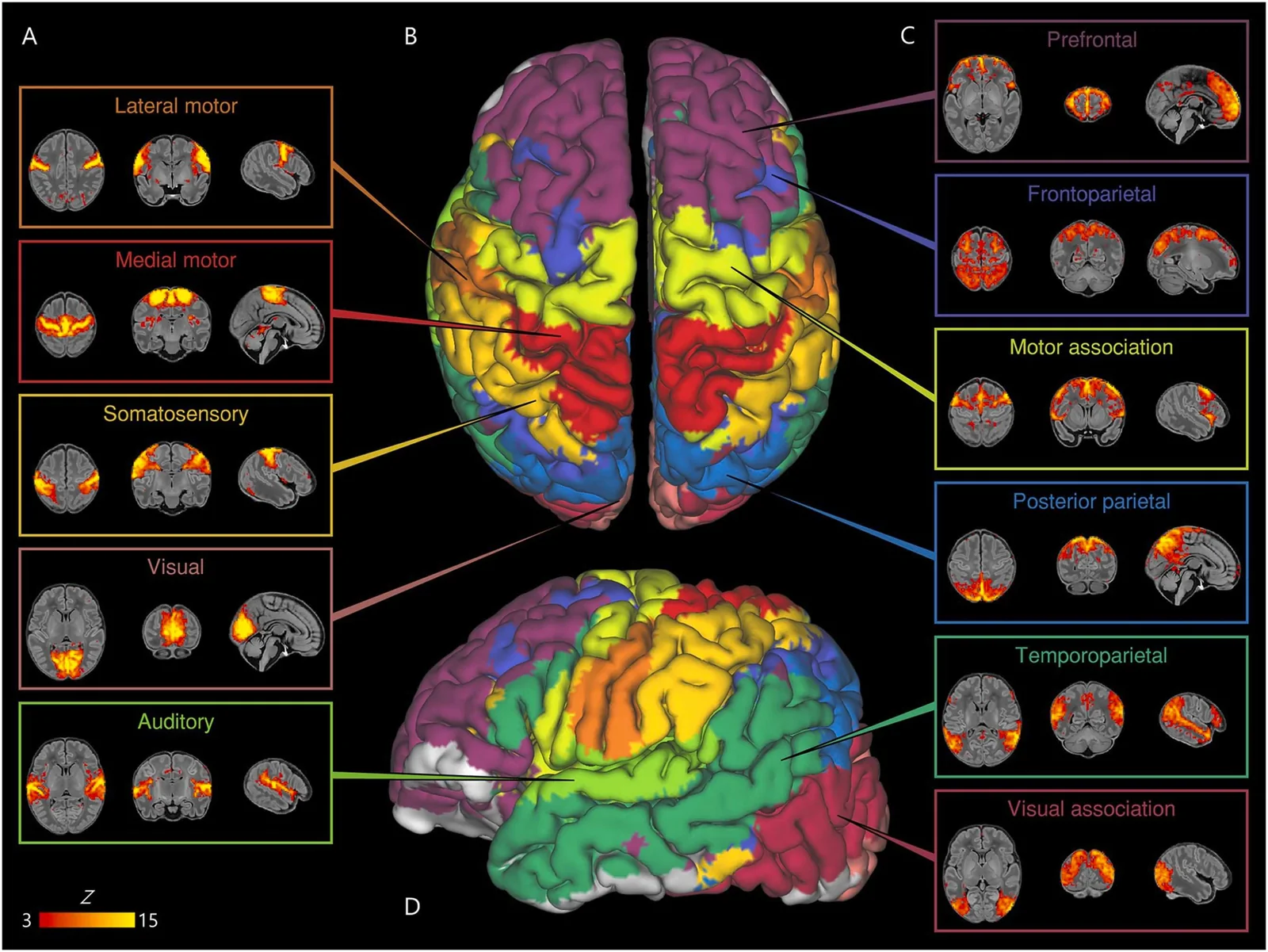
Resting-state functional networks identified in the neonatal brain at term-equivalent age. Group independent component analysis of spontaneous blood oxygen level-dependent (BOLD) activity reveals a spatially differentiated functional architecture by the neonatal period. Primary networks, including sensorimotor, visual and auditory systems, and higher-order association networks, including frontoparietal and temporoparietal systems, are shown. These maps illustrate that large-scale functional organization is detectable in the neonatal brain, whereas higher-order systems remain less mature and continue to undergo refinement. Reproduced from Eyre et al. (2021), *Brain*, 144, 2199–2213, under the terms of the Creative Commons Attribution License (CC BY 4.0).

Primary systems show particularly prominent age-related differences during the perinatal period, whereas association systems generally develop over a more extended timescale. During the first postnatal month, age-related connectivity patterns were detectable throughout the brain but were more pronounced in sensorimotor systems than in most association networks, with the cingulo-opercular network representing an important exception (Nielsen et al., 2023). Nevertheless, rudimentary organization of higher-order networks can already be detected during the fetal and neonatal periods (Molloy and Saygin, 2022, Rajasilta et al., 2020, Thomason et al., 2015).At term-equivalent age, group independent component analysis identified primary sensorimotor, visual, and auditory networks, together with association components involving prefrontal, frontoparietal, temporoparietal, posterior parietal, motor-association, and visual-association cortices (Eyre et al., 2021)(Fig. 1).

Using a different analytical framework, Sylvester et al. (2023) found network-selective long-range functional connections in neonatal precursors of the default mode network (DMN), frontoparietal network (FPN), and dorsal attention network (DAN). This long-range selectivity was weaker than that observed in older children and adults, while local connectivity was stronger in neonates. Thus, a latent distributed network architecture is detectable near birth, but its expression can be obscured by prominent short-range connectivity and can vary with network-detection methods, surface registration, and the amount of usable data.

At the same time, patterns of network interaction also began to differentiate. Inter-network coupling between the DMN and FPN increased with gestational age, whereas connectivity between the salience network (SN) and either the DMN or FPN decreased (Scheinost et al., 2024). The anticorrelation between the default mode network (DMN) and dorsal attention network (DAN), a classic functional signature of competition between internally and externally oriented cognitive domains in the adult brain, had begun to emerge by term, as reflected in the initial development of relatively low inter-network coupling between the two networks (H. Hu et al., 2022). Overall, these findings suggest that local specialization, cross-network integration, and inter-network differentiation may begin early in life (Scheinost et al., 2024).

#### 2.1.4 Spatiotemporal gradients and sex-related variation

Studies conducted at earlier gestational ages more often reported sparse interhemispheric connections or incomplete bilateral networks, whereas functional coupling between homologous brain regions gradually strengthened as fetal development progressed (Dall’Orso et al., 2022, Emerson et al., 2016). Studies of the sensorimotor network showed that connectivity between bilateral homologous limb-representation regions increased with gestational age between 32 and 45 weeks and exhibited a near-adult spatial organization around term (Dall’Orso et al., 2022). A longitudinal study of the language network similarly found that homologous connectivity between the left and right Broca’s and Wernicke’s areas increased markedly from 30 weeks of gestation to the first postnatal month, whereas intrahemispheric connectivity between language regions did not show a comparable increase during the same period (Scheinost et al., 2022a). In addition, the developmental timing of functional connectivity (FC) was related to anatomical distance: homologous connections near the midline emerged earlier, whereas more lateral homologous connections separated by greater anatomical distances emerged later (De Asis-Cruz et al., 2020). Early networks were generally characterized by stronger interhemispheric connectivity and relatively limited intrahemispheric integration (Smyser et al., 2010).

Taken together, multiple rs-fMRI studies suggest that fetal functional connectivity may exhibit an overall posterior-to-anterior spatial gradient. During mid-gestation, more prominent connections were located mainly in occipital/visual regions, parts of the temporal lobe, and other posterior and medial sensory-related systems. With advancing gestational age, the involvement of frontal and parietal regions and long-range cross-system connections became increasingly apparent (Jakab et al., 2014, Thomason et al., 2015). Near term, primary networks, including the sensorimotor, visual, and auditory networks, already showed adult-like spatial configurations, whereas the posterior parietal, frontoparietal, prefrontal, and visual association networks continued to show PMA-related spatial expansion, and within-network connectivity strength continued to increase in some networks (Eyre et al., 2021). However, Taymourtash et al. (2023) did not identify a significant maturational sequence across different cerebral lobes. This posterior-to-anterior gradient is therefore better understood as an overall spatial tendency observed across studies rather than a fixed linear sequence shared by all functional networks.

Sex-related differences have also been reported in some early functional networks. During the fetal period, some associations between FC and gestational age differed by sex, involving systems such as posterior cingulate-temporal and frontal-cerebellar connectivity (Wheelock et al., 2019). Near term, female infants showed relatively stronger functional connectivity in infero-occipitotemporal visual association regions and the left posterior superior temporal sulcus (Eyre et al., 2021, Mancuso et al., 2025), which may be related to early social visual preferences. Fenske et al. (2025) further found that some associations between early FC and later behavior differed by sex, although no independent main effect of sex was observed for the corresponding connections themselves. Current findings therefore suggest that sex may be an important source of variation in early functional organization and its behavioral associations. Whether these group-level differences predict subsequent cognitive or socioemotional development remains to be established through longitudinal studies and validation in independent cohorts.

### 2.2 Infancy: the first postnatal year

Building on the functional organization established prenatally, large-scale brain networks continue to reorganize and become more finely differentiated during the first postnatal year. Longitudinal rs-fMRI studies indicate partially overlapping age-related changes in primary sensorimotor systems, higher-order association networks and their interactions, alongside the rapid expansion of infants’ sensory, motor and social experience (Gao et al., 2015, Liu et al., 2023; F. Wang et al., 2023; Wen et al., 2019).

#### 2.2.1 Topological refinement and spatial reorganization

Topological refinement during infancy appears to involve the concurrent progression of modular differentiation, hub redistribution and integration across spatial scales. Using rs-fMRI data acquired during the neonatal period and at 3, 6, 9 and 12 months of age, Wen et al. (2019) found that the infant functional brain network was progressively subdivided, with the number of modules increasing from four in neonates to eight at 12 months. Both within-module and between-module functional connectivity increased during this period.

The topological roles of functional hubs also changed during the first year. Connector hubs, which link different modules, became increasingly prominent, whereas provincial hubs, which primarily support communication within a single module, became less prevalent. Some regions transitioned from non-hub regions to hubs, whereas others shifted from provincial to connector hubs. This pattern suggests that the differentiation of local functional systems and the development of communication between modules can proceed concurrently.

A study covering the first 1000 days further showed that high-connectivity hubs were predominantly located in the sensorimotor system during the perinatal period. During the postnatal period, hubs were increasingly observed in the dorsal attention and visual systems, whereas the default-mode system became more prominent by approximately 2 years of age (Li et al., 2024),its results should be interpreted as complementary to the connector-hub and provincial-hub findings reported by Wen et al. (2019).

Infant functional connectivity remains dominated by short-range connections, together with a smaller number of long-range connections that are preferentially associated with hub regions. Li et al. (2024) found that short- to medium-range functional connectivity strength increased before 1 year of age and reached its peak at approximately 10–11 months, whereas long-range connectivity peaked later, at approximately 15 months. Short- to medium-range connectivity and local clustering subsequently declined, while long-range connections continued to develop, consistent with a gradual shift from local specialization toward more distributed integration.

A multivariate age-classification study provided related evidence for spatial reorganization between 6 and 12 months. Stronger posterior left–right connections contributed more to the classification of 6-month-old infants, whereas stronger anterior–posterior connections contributed more to the classification of 12-month-old infants (Pruett et al., 2015). The authors noted that this pattern was superficially consistent with the reported posterior-to-anterior sequence of white-matter maturation.

#### 2.2.2 Higher-order network organization and inter-network differentiation

Age-related changes are not synchronized across functional networks. Studies using fine-grained, infant-specific functional parcellations further showed that changes in network organization were generally greater between 3 and 9 months than between 9 and 24 months. After 9 months, primary sensory networks gradually developed relatively adult-like spatial patterns, whereas higher-order networks, including the default mode, frontoparietal, and dorsal attention networks, underwent marked organizational changes between 3 and 9 months and continued to be refined between 12 and 24 months. Even at 24 months, the default mode and frontoparietal networks still lacked stable and strong cross-lobar connections (F. Wang et al., 2023). However, because this study used an accelerated longitudinal design in which the infants assessed at different ages were generally not the same individuals, its findings primarily reflect age-related changes at the group level.

During the same period, key patterns of inter-network interaction also became more firmly established. A negative correlation between the DMN and SN was already present at 3 months (Li et al., 2023), and interactions between the DMN and DAN increased significantly between 6 and 12 months (Gao et al., 2013). Longitudinal observations of language-related regions showed that interhemispheric connectivity between the bilateral inferior frontal and superior temporal gyri increased during the first year and peaked at approximately 11.5 months (Emerson et al., 2016).

A repeated-measures longitudinal study suggested that infant functional connectivity does not follow a single, uniform maturational trajectory. Among 90 infants who completed scans during the neonatal period and at 1 and 2 years of age, logarithmic growth trajectories were the most common, particularly in higher-order association regions and their connections with distant brain regions. Pruning-like and transient trajectories were also observed, with different trajectories concentrated in different brain regions (Liu et al., 2023). Overall, current evidence supports a hierarchical framework in which primary systems establish relatively stable functional organization earlier, whereas higher-order association networks undergo more prolonged refinement; however, this hierarchy is overlapping and regionally heterogeneous.

#### 2.2.3 Development of subcortical and cerebellar–cortical functional organization

Early motor behaviour in infancy does not rely exclusively on the cortical motor system. At birth and during the first few postnatal months, descending control by the motor cortex remains immature, and the organization and generation of early movements may depend more heavily on brainstem-centred subcortical mechanisms. With development, the cortex and subcortical motor networks gradually achieve more complete functional integration (Blumberg and Adolph, 2023).

Cerebellar–cortical functional coupling also develops from a relatively diffuse organization towards a more differentiated spatial pattern. A study based on 1017 scans, with up to six repeated measurements per individual, found that functional connectivity between the cerebellum and several higher-order networks was already detectable at birth and generally increased with age. At the same time, cerebellar functional topology became progressively more focal, evolving from a relatively diffuse spatial distribution early in development, whereas the relative extent of the primary sensorimotor networks within the cerebellum decreased with age (Lyu et al., 2025).

Another mixed longitudinal study showed that, during the neonatal period, cerebellar functional connectivity was concentrated mainly in sensorimotor, visual and basal ganglia-related systems. From the neonatal period to 1 year of age, connectivity between the cerebellum and higher-order regions, including the prefrontal cortex, increased, whereas connectivity with some primary networks relatively decreased; no changes of comparable magnitude were observed between 1 and 2 years of age (Tikoo et al., 2025). Together, these findings support the gradual differentiation of cerebellar–cortical functional coupling during infancy and increasing coordination with higher-order association regions. However, studies do not fully agree on when connectivity with higher-order networks emerges, possibly owing to differences in ICA model order, network definitions, methods for calculating connectivity and cohort composition.

In addition, the temporal and frequency characteristics of the infant BOLD signal show age-related changes. At the level of the BOLD signal spectrum, a longitudinal study found that the peak of spontaneous BOLD fluctuations shifted overall towards higher frequencies during the first postnatal year and gradually acquired spectral characteristics more similar to those of adults (Alcauter et al., 2015). This spectral shift may accompany multiple neurodevelopmental and neurovascular changes during infancy.

## 3 Preterm birth-related differences in functional network development

Available rs-fMRI evidence indicates that functional network organization in infants born preterm differs from that of term-born infants assessed at a comparable postmenstrual age (PMA), but these differences do not reflect a uniform developmental delay across all functional systems. In infants without major brain injury, Dall’Orso et al. (2022) found that developmental changes in sensorimotor connectivity were more strongly related to age at scan than to birth status, and that the overall sensorimotor trajectory was not significantly altered by preterm birth. This relative preservation contrasts with broader differences reported in thalamocortical and higher-order association systems (Derman and Ferradal, 2025, Smyser et al., 2010). These early group-level differences may be relevant to later neurodevelopmental vulnerability, although their individual prognostic significance remains uncertain.

Model-based analyses further suggest that some network properties may follow different age-related trajectories after preterm birth. Using data from 332 term-born and 115 preterm neonates, López-Guerrero and Alcauter, 2025 reported nonlinear trajectories for several graph-theoretical measures in preterm neonates, whereas the corresponding patterns in term-born neonates were closer to linear. However, repeated scans were available for only a subset of the preterm participants, limiting conclusions about within-individual developmental trajectories.

Evidence concerning the timing of these differences is also mixed. Compared with gestational-age-matched fetuses remaining in utero, ex utero very preterm infants showed stronger connectivity in selected superior temporal, hippocampal and occipital regions, suggesting that extrauterine exposure may modify specific sensory- and stress-related networks (De Asis-Cruz et al., 2020). At the same time, fetal rs-fMRI studies indicate that some differences may precede delivery. Fetuses classified as being at elevated risk of preterm birth showed fewer estimated functional connections in a small prospective cohort (Miglioli et al., 2024), while fetuses who were subsequently born preterm exhibited weaker systems-level functional connectivity, particularly in a left-hemisphere region implicated in later language processing (Thomason et al., 2017). Preterm-related network differences may therefore reflect both prenatal vulnerability and postnatal extrauterine exposure.

### 3.1 Differences in global network topology

Although functional networks in very preterm infants retain small-world topology and modular organization at term-equivalent age (TEA), their balance between segregation and integration differs from that of term-born neonates. In a comparison of 66 very preterm and 66 term-born infants, Bouyssi-Kobar et al. (2019) found lower clustering, local efficiency, global efficiency and modularity, together with longer characteristic path length, in the very preterm group. These findings indicated reduced functional segregation and integration despite preservation of the overall small-world architecture.

A larger dHCP analysis similarly found that lower gestational age at birth was associated with lower overall connectivity strength, clustering coefficient and global efficiency, as well as longer characteristic path length, at TEA (López-Guerrero and Alcauter, 2025). In a separate study of 24 very preterm and 51 term-born infants, Gozdas et al. (2018) reported a lower rich-club coefficient and assortativity but higher small-worldness in the preterm group, with no significant difference in modularity.

Related differences have also been observed in cerebellar systems. Herzmann et al. (2019) found that the overall topology of intra-cerebellar and cerebellar–cortical connectivity was identifiable and broadly similar in very preterm and term-born infants at TEA, whereas the magnitude of the corresponding functional correlations was lower in the preterm group. Thus, preterm birth may affect connectivity strength without necessarily eliminating the basic spatial organization of the network.

### 3.2 Higher-order network development after preterm birth

Preterm birth-related differences are particularly pronounced in higher-order association networks (He and Parikh, 2016, Smyser et al., 2016), and lower gestational age at birth is associated with more marked reductions in functional connectivity (Eyre et al., 2021). Importantly, higher-order networks are not entirely “absent” at term-equivalent age (TEA), and functional networks in preterm infants continue to develop after birth.

Hu et al. (2022) found that the default mode network (DMN), dorsal attention network (DAN) and executive control network (ECN) could be identified as relatively distinct networks in both term-born neonates and preterm infants at TEA. However, compared with term-born neonates, preterm infants at TEA exhibited lower within-network connectivity in the DMN and DAN and higher between-network connectivity between the DAN and ECN, indicating weaker within-network synchrony and less functional differentiation between the DAN and ECN. By contrast, among preterm infants scanned before TEA, only the DAN showed clear within-network differentiation (Smyser et al., 2010), and typical interactions between networks were not yet evident (Della Rosa et al., 2021).

Using 66 valid scans acquired from very preterm infants at 32, 39 and 52 weeks of postmenstrual age (PMA), He and Parikh (2016) found that connectivity within the auditory, somatosensory, visual, subcortical grey matter, executive control and frontoparietal networks increased with age. Taken together, even at TEA, connectivity among higher-order networks in preterm infants may remain atypical (Herzmann et al., 2019), and network differentiation may remain less developed than in term-born infants.

### 3.3 Regional increases in connectivity, sensory-related reorganization, and functional lateralization

Preterm birth is not uniformly associated with reduced functional connectivity. Relative increases have been observed in selected sensory and sensory-association regions. Compared with gestational-age-matched fetuses remaining in utero, ex utero very preterm infants showed stronger connectivity involving the superior temporal cortex, hippocampus and occipital regions (De Asis-Cruz et al., 2020). At TEA, very preterm infants also showed greater connection density within an occipital module (Bouyssi-Kobar et al., 2019) and increased connectivity of the bilateral superior parietal lobules within the lateral motor network(Eyre et al., 2021).

Thalamocortical organization shows a similarly nonuniform pattern. Greater prematurity was associated with stronger connectivity between the thalamus and lateral primary sensory cortices but weaker connectivity between the thalamus and prefrontal, insular and anterior cingulate regions (Toulmin et al., 2015). Together, these findings are consistent with an imbalance between sensory-related organization and the development of higher-order integrative systems after preterm birth.

Complementary evidence comes from regional measures of spontaneous BOLD activity. Feng et al. (2024) also found that, compared with 14 term-born infants, 15 preterm infants showed higher amplitude of low-frequency fluctuations (ALFF) in the left precuneus, the orbital part of the superior frontal gyrus, and the calcarine cortex. The developmental significance of these regional ALFF increases remains unclear, but they may be related to the developmental coordination difficulties commonly observed following preterm birth.

Preterm birth has additionally been associated with differences in functional lateralization. Kwon et al. (2015) found that very preterm infants showed less lateralized connectivity in several language-related frontotemporal regions at TEA than term-born infants, which may be related to the risk of later language delay, although the study did not directly examine language outcomes. Future longitudinal studies incorporating repeated scans, standardized processing pipelines, and independent outcome validation are needed to determine whether these imaging differences represent stable individual developmental trajectories and clinically meaningful predictive indicators.

## 4 Environmental sensitivity and plasticity of early functional networks

Functional networks during the fetal and infant periods undergo rapid formation, differentiation and integration. In a study of 268 twin and singleton neonates, only 6 of 36 functional-connectivity phenotypes had heritability estimates above 0.10, and none reached statistical significance (Blanchett et al., 2023). These findings suggest that the detectable genetic contribution to neonatal functional connectivity was limited in this cohort and that early network organization may be sensitive to sensory experience, maternal state, the social environment and perinatal clinical exposures. Table 1 summarizes representative exposures and interventions associated with differences in early functional networks.

### 4.1 Sensory experience and social interaction

Studies of music-based interventions in preterm infants provide some of the more direct evidence for experience-dependent plasticity of early functional networks. In a randomized controlled pilot study, 82 preterm infants were assigned to intervention or control groups, but only 40 contributed data to the MRI analysis. Infants who received creative music therapy showed shorter thalamocortical processing delays and stronger functional integration involving parts of the prefrontal cortex, supplementary motor area and temporal cortex (Haslbeck et al., 2020). In another rs-fMRI study, the music-exposure group showed stronger connectivity between the salience network and auditory, sensorimotor, superior frontal, thalamic and posterior cingulate/precuneus systems, with some network features more similar to those of term-born neonates (Lordier et al., 2019b). A subsequent longitudinal study repeatedly scanned 43 preterm infants at approximately 33 and 40 weeks of gestational age. Several connections changed with age during this interval, but the music-intervention group showed a greater change than the comparison group only in connectivity between the thalamus/brainstem and the salience network (Van Der Veek et al., 2024).

Evidence from task-based fMRI also suggests that postnatal auditory experience is detectable early. Within the first postnatal weeks, the non-primary auditory cortex showed some degree of selective response to music (Kosakowski et al., 2023). In a separate task-based fMRI study using connectivity analyses, familiar music was related to coupling between the auditory cortex, thalamus and dorsal striatum in preterm infants (Lordier et al., 2019a). King et al. (2021) found that the frequency of adult-infant conversational turns was associated with language-network connectivity in 5- to 8-month-old infants. In their analysis, more conversational turns were related to lower connectivity within posterior temporal language circuits, indicating that the direction and meaning of this association are not equivalent to a simple increase in connectivity. The finding nevertheless suggests that contingent, interactive language experience may be related to early language-network organization in ways that cannot be reduced to the amount of adult speech heard by the infant.

Longer-term follow-up evidence remains limited. In a non-randomized feasibility study, the creative music-therapy and usual-care groups did not differ significantly in cognitive, language or motor outcomes at 2 years. At 5 years, some cognitive scores were numerically higher in the music-therapy group, but between-group differences were not statistically significant; the study was small and had substantial attrition (Haslbeck et al., 2023). Taken together, these studies suggest that structured auditory input may modulate selected early functional circuits, but they do not establish that such input improves long-term neurodevelopmental outcomes.

### 4.2 Maternal psychological state and social environment

Existing rs-fMRI studies indicate that maternal psychological state and the surrounding social environment are associated with differences in early functional connectivity. Reported associations involve limbic and subcortical structures, including the amygdala, hippocampus and thalamus, as well as higher-order systems such as the default mode, salience and frontoparietal networks. The implicated regions, connection directions and developmental stages differ substantially across studies (Hill et al., 2025, Na et al., 2023, Rajasilta et al., 2023, Scheinost et al., 2020, Thomason et al., 2021a). The amygdala is a key node in the evaluation of emotional salience and emotion regulation and has therefore been used frequently as a seed region in this literature.

Associations between maternal psychological state and offspring connectivity can already be observed during the fetal period. Maternal prenatal negative affect and stress have been associated with differences in fetal frontoparietal, striatal and temporoparietal connectivity (Thomason et al., 2021a). In a cohort consisting mainly of pregnant adolescents, higher multidimensional distress in late pregnancy was associated with weaker fetal hippocampal-cingulate connectivity and stronger hippocampal-temporal connectivity; some of these connections were also related to memory performance at 4 months of age (Scheinost et al., 2020). A repeated-measures fetal-neonatal study further found that higher composite scores for maternal stress, anxiety and depressive symptoms were associated with lower within-network connectivity in the salience network (Scheinost et al., 2024). However, the mental-health analysis included only 29 mother-infant dyads, and the default-mode and frontoparietal findings did not reach the prespecified evidential threshold. These results should therefore be regarded as exploratory (Scheinost et al., 2024).

Associations with maternal psychological state have also been reported in neonates and infants. Late-gestation subclinical depressive symptoms were associated with lower within-frontal connectivity and lower connectivity between frontal or temporal regions and the occipital cortex in neonates (Na et al., 2023). A small study reported differences in neonatal medial prefrontal local activity and long-range connectivity in relation to maternal psychological symptoms (Rajasilta et al., 2023). In a seed-based study, higher prenatal depressive symptoms were associated with stronger connectivity between the left amygdala and the insula, anterior cingulate cortex and medial prefrontal cortex at 6 months of age (Qiu et al., 2015). The timing and trajectory of perinatal stress may also matter. A rising or peak pattern of late-pregnancy stress was associated with stronger neonatal amygdala-anterior insula and amygdala-ventromedial prefrontal connectivity, although these imaging findings have not yet been independently validated (Marr et al., 2023). Anxiety measured at different stages of pregnancy was associated with lower neonatal amygdala-fusiform connectivity and higher amygdala-thalamic connectivity, respectively (Hill et al., 2025). Together, these findings suggest that the timing and longitudinal pattern of exposure may influence the connectivity pattern observed at birth.

Caregiver psychological state after birth may also be related to the organization of infant emotion-related networks. In a discovery sample and an independent replication sample, Phillips et al. (2021) found that stronger amygdala-salience-network connectivity and weaker amygdala-executive-control-network connectivity in 3-month-old infants statistically mediated the association between caregiver postpartum depression or anxiety and reduced infant smiling. This result supports a possible role for infant connectivity in the statistical association between caregiver psychological state and early emotional behaviour; it does not establish a causal pathway. Maternal adverse childhood experiences have likewise been associated with offspring connectivity differences. Maternal-reported childhood emotional neglect was associated with stronger neonatal amygdala-ventromedial prefrontal or amygdala-dorsal anterior cingulate connectivity, whereas maternal childhood maltreatment was associated with differences in fetal amygdala connectivity with frontal, premotor and brainstem regions (Hendrix et al., 2021, Van Den Heuvel et al., 2023). Because these experiences were assessed mainly by retrospective maternal report and may co-occur with current psychological state and social conditions, the pathway linking them to offspring connectivity remains unresolved.

Social conditions at the family and community levels may be associated with broader aspects of early network organization. In 261 term-born neonates, prenatal social disadvantage was associated with differences in whole-brain functional connectivity at birth, with effects involving frontoparietal, ventral-attention, dorsal-attention and sensorimotor networks, as well as subcortical structures including the amygdala; the findings were replicated in an independent sample (Nielsen et al., 2024). A longitudinal analysis from the same research programme further found that prenatal social disadvantage was associated with a steeper age-related increase in cortical network segregation from birth to 3 years, with effects distributed along the sensorimotor-to-association cortical hierarchy (Tooley et al., 2024). Community safety represents another dimension of the social environment. Higher levels of property and violent crime were associated with weaker neonatal thalamus-anterior default-mode-network connectivity, and violent crime was also associated with weaker amygdala-hippocampal connectivity; maternal psychosocial stress statistically mediated part of these associations (Brady et al., 2022). Lower socioeconomic status was associated with differences in neonatal striatal and ventrolateral prefrontal connectivity. Some frontostriatal connections showed statistical mediation of the association between socioeconomic status and behavioural inhibition or externalizing symptoms at 2 years, although the behavioural follow-up sample was small and requires replication (Ramphal et al., 2020). More recent prospective work suggests that the association between prenatal adversity and later executive function does not necessarily first manifest as a stable rs-fMRI network effect (Lean et al., 2026). Early social conditions may also operate through white matter, caregiving and other physiological pathways, making a single-mechanism model based on a small number of significant functional connections unlikely to be sufficient.

Evidence concerning the neural correlates of racial discrimination and related social stressors remains limited. Two small studies reported associations between maternal experiences of discrimination or racism and neonatal amygdala or hippocampal connectivity, involving the prefrontal cortex, fusiform gyrus, visual cortex and thalamus (Kral et al., 2024, Spann et al., 2024). These findings suggest that structural and interpersonal social stressors may be related to limbic-sensory and limbic-cortical connectivity at birth. However, given the small samples, reliance on self-reported exposure measures and lack of later behavioural validation, they should currently be regarded as preliminary associations.

Overall, available studies provide relatively consistent evidence that maternal psychological state and social conditions are associated with differences in early offspring functional networks. The amygdala, hippocampus, thalamus, salience network and frontoparietal systems are repeatedly implicated. This concentration may reflect heightened environmental sensitivity of limbic and higher-order regulatory systems during the perinatal period.

### 4.3 Metabolic, immune and perinatal exposures

Maternal metabolic and immune status, perinatal medication, substance and environmental exposures, and early nutrition have all been associated with differences in early or later brain organization. Several small studies have linked higher prepregnancy body mass index (BMI) to altered neonatal connectivity and graph-theoretical measures (Rajasilta et al., 2021, Salzwedel et al., 2019). In 45 neonatal rs-fMRI datasets, higher maternal BMI was associated with stronger whole-brain connectivity of the left thalamus and stronger local connectivity of both thalami, but with weaker connectivity in parts of the prefrontal-thalamic system (Spann et al., 2020). In a separate study of 21 mother-infant pairs, higher prepregnancy BMI was associated with higher regional homogeneity (ReHo) in the neonatal left superior frontal gyrus and weaker long-range connectivity involving that region (Rajasilta et al., 2021). A larger dynamic analysis of dHCP data further linked higher prepregnancy BMI to a lower probability of occupying a frontoparietal functional state and to fewer transitions from sensorimotor to frontal states (Mariani Wigley et al., 2026). These findings suggest an association between maternal metabolic status and early thalamic and frontal organization, but they do not identify a single underlying mechanism.

Maternal interleukin-6 (IL-6) concentrations during pregnancy have been associated with neonatal salience-network connectivity and amygdala-related measures. Two analyses from the same longitudinal cohort found that higher maternal IL-6 was associated with differences in working memory at 2 years (Rudolph et al., 2018) and that neonatal amygdala-related measures statistically mediated the association between maternal IL-6 and impulse-control outcomes at 2 years (Graham et al., 2018). The mediation model should not be interpreted as proof that IL-6 caused poorer impulse control. Spann et al. (2018) further reported that late-gestation IL-6 and C-reactive protein concentrations were associated with neonatal salience-network connectivity differences involving the insula, dorsal anterior cingulate and medial prefrontal cortex. Both inflammatory markers were positively associated with cognitive scores at 14 months, but neonatal connectivity did not show a significant mediation effect. Animal work provides complementary mechanistic evidence: in a mouse model of sterile chorioamnionitis, IL-6-receptor blockade reduced IL-1alpha-induced preterm birth and neonatal mortality and improved growth, neuromotor performance and immune homeostasis (Farias-Jofre et al., 2023).

Studies of perinatal medication exposure have produced complex results. In a non-randomized study, very preterm infants who received late systemic dexamethasone treatment showed stronger dorsal-attention-to-visual and default-mode-to-auditory connectivity at term-equivalent age than untreated infants; some of these connections were also associated with developmental scores at 3 months (Jia et al., 2023). Because dexamethasone treatment is related to clinical indications such as mechanical ventilation and bronchopulmonary dysplasia, the findings are vulnerable to confounding by indication and do not establish a direct neuroprotective effect. In a randomized placebo-controlled trial, prenatal magnesium-sulfate exposure was associated with higher voxelwise connectivity in temporo-occipital regions and deep grey matter, as well as higher clustering, local-efficiency and global-efficiency measures at term-equivalent age (Ufkes et al., 2024). The analyzed sample was small, and these connectivity differences cannot by themselves be interpreted as evidence of clinical benefit.

Small studies of specific prenatal exposures have reported differences in trajectories of interhemispheric and posterior-cingulate-to-lateral-prefrontal connectivity in relation to fetal lead exposure, differences in fetal hippocampal networks in relation to prenatal cannabis exposure, and altered spatial distributions of functional hubs in relation to prenatal alcohol exposure (Roos et al., 2021, Thomason et al., 2021b, Thomason et al., 2019). Studies of prenatal opioid exposure have examined amygdala-prefrontal connectivity, thalamocortical connectivity, whole-brain network topology and the spatial organization of visual and executive-control networks (Merhar et al., 2021, Radhakrishnan et al., 2022a, Radhakrishnan et al., 2022b, Radhakrishnan et al., 2021). In a whole-brain rs-fMRI study of 133 neonates, prenatal exposure to nicotine, alcohol, cannabis, cocaine, opioids or selective serotonin-reuptake inhibitors was associated with exposure-specific connectivity patterns that were concentrated overall in higher-order networks; some affected connections were also associated with cognitive, language and motor scores at 3 months (Salzwedel et al., 2020). Most of these studies were small, and opioid exposure frequently co-occurred with tobacco use, other medications, maternal psychological symptoms and social disadvantage, making it difficult to isolate the independent contribution of any single substance.

Evidence concerning environmental chemical exposure is also emerging. In 61 neonates, higher prenatal exposure to fine particulate matter (PM2.5) was associated with stronger local Iso-Distant Average Correlation (IDAC), with effects concentrated in sensorimotor regions and showing distance dependence in premotor and supplementary motor areas (Blanco-Hinojo et al., 2026). The authors noted that stronger local connectivity could reflect delayed differentiation of primary systems or accelerated local organization within the sensorimotor hierarchy. Because the study did not include later behavioural follow-up, its developmental significance remains unknown.

The relationship between early nutrition and brain development may have clinical relevance (Belfort and Inder, 2022, Zhang et al., 2022). For example, in 56 children born preterm and assessed at 7 years of age, higher protein intake during the first week and first month after birth was associated with stronger thalamus-default-mode-network connectivity; stronger connectivity was in turn associated with higher processing-speed and visual-processing scores (Duerden et al., 2021).

Overall, metabolic and immune status, medications, substance and environmental exposures and nutrition have each been associated with differences in early or later functional brain organization, but the strength and design of the evidence vary substantially. With the exception of the magnesium-sulfate study, most evidence comes from small observational cohorts. Further validation is needed in future studies.

### 4.4 NICU clinical exposures and modifiable care environments

For preterm infants and other high-risk neonates, the neonatal intensive care unit (NICU) provides essential life support but also introduces repeated invasive procedures, pain-related stimulation and complex sensory exposures. Cook et al.(2023) compared 148 extremely or very preterm infants with 99 term-born infants and examined skin-breaking procedures in the preterm group, including heel lances, venepuncture and intravenous catheter placement, in relation to functional connectivity at term-equivalent age. A greater number of skin-breaking procedures was associated with stronger within-cerebellar connectivity and stronger connectivity between the cerebellum and association, limbic and paralimbic regions. At 18 months, a higher procedure count was also associated with greater autism-related risk on the M-CHAT-R and lower gross-motor, fine-motor and expressive-language scores. Because only approximately 45 infants contributed both imaging and neurodevelopmental follow-up, and because procedure counts were strongly related to illness severity and monitoring requirements, the study could not adequately separate the contribution of pain-related procedures from the burden of underlying illness. Analgesia, kangaroo care, swaddling and pacifier use were not systematically quantified. The findings therefore support an association between repeated invasive procedures and differences in preterm cerebellar organization and later development, but they do not demonstrate that pain exposure directly caused these differences.

Neuroimaging findings attributed to NICU exposure also need to be distinguished from pre-existing disease and physiological state. De Asis-Cruz et al. (2018) compared 30 neonates with complex congenital heart disease (CHD) and 82 healthy term-born neonates before cardiac surgery. Both groups showed small-world and rich-club organization, and global efficiency and cost efficiency were broadly comparable. However, the CHD group showed a subnetwork with weaker connectivity, involving the basal ganglia, thalamus and brainstem and their connections with frontal, parietal and temporal cortices. The CHD group also had fewer rich-club nodes and lower connection density among those nodes. Because these differences were present before surgery, they may relate to prenatal and early postnatal haemodynamic abnormalities, disease burden or differences in brain development rather than to postoperative treatment.

Potentially modifiable care-related factors extend beyond the NICU. In a longitudinal study, higher prenatal community-crime exposure was associated with more externalizing symptoms between 1 and 2 years of age, but neonatal functional connectivity did not statistically mediate the association between crime exposure and externalizing symptoms (Brady et al., 2024b). By contrast, lower positive engagement and sensitivity and higher negative attention during parent-child interaction at 1 year statistically mediated the association between prenatal crime exposure and externalizing symptoms at 2 years. These findings identify caregiving behaviour as a factor warranting further investigation in intervention studies.

Overall, early neonatal functional-network differences may reflect the combined influence of prematurity and underlying disease, invasive medical procedures, physiological instability and the postnatal caregiving environment. Future studies should prospectively record the timing and frequency of clinical exposures and pain-management measures, adjust for illness severity, use repeated rs-fMRI and long-term behavioural follow-up, and apply randomized or quasi-experimental designs to test whether specific care interventions can alter the trajectory of functional-network development.

## 5 Potential for clinical translation

**Fig. 2:**
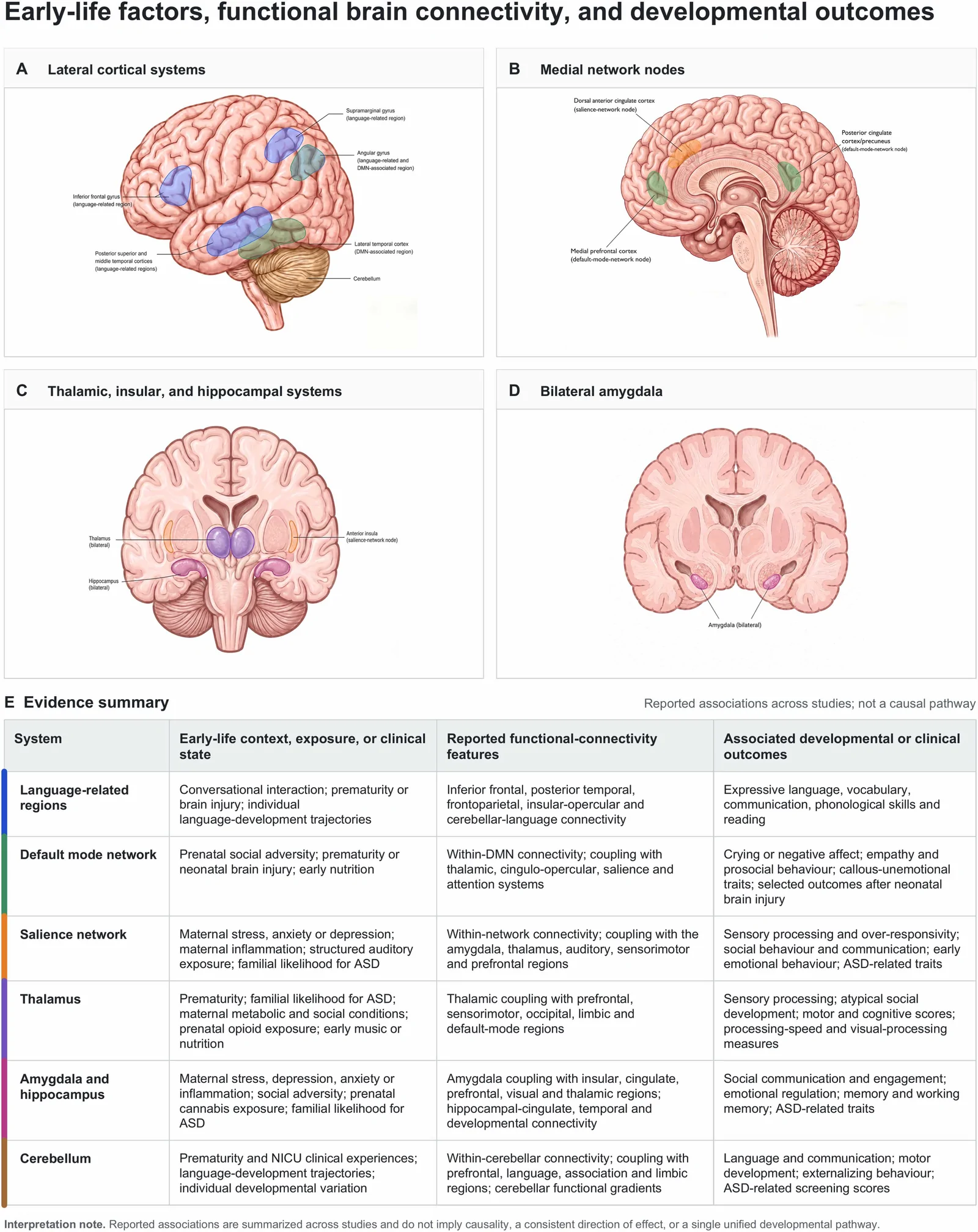
Early-life factors, functional brain connectivity, and developmental outcomes. (A-D) Schematic lateral, medial, and coronal views show representative cortical, subcortical, and cerebellar regions discussed in early-life functional-connectivity studies. Panel A shows language-related lateral cortical regions, default-mode-network-associated lateral temporal regions, and the cerebellum; panel B shows medial default mode and salience network nodes; panel C shows the bilateral thalamus, anterior insula, and hippocampus; and panel D shows the bilateral amygdala. Highlighted regions indicate approximate anatomical locations rather than atlas parcels or activation maps. (E) Representative evidence is organized by functional system, early-life context, exposure or clinical state, reported functional-connectivity feature, and associated developmental or clinical outcome. The figure summarizes group-level associations across heterogeneous fetal, neonatal, and infant studies and does not imply causality, a consistent direction of effect, validated biomarkers, or individual-level prediction. Abbreviations: ASD, autism spectrum disorder; DMN, default mode network; NICU, neonatal intensive care unit.

Beyond characterizing the developmental trajectories of early brain networks, resting-state functional magnetic resonance imaging (rs-fMRI) studies have detected functional connectivity differences before behavioural symptoms become apparent and reported prospective associations between these differences and subsequent neurodevelopmental outcomes. Early functional connectivity may therefore provide candidate imaging indicators for risk identification and prognostic assessment (Fig. 2). However, the existing evidence is derived primarily from comparisons between risk groups, brain-behaviour associations, or prediction analyses conducted within a single sample, and has not yet adequately demonstrated individual-level diagnostic accuracy, cross-cohort reproducibility, or incremental value relative to conventional clinical indicators. Table 2 summarizes functional connectivity characteristics in early life and their associations with subsequent neurodevelopmental outcomes.

### 5.1 Early network features in infants at high familial likelihood for ASD

Studies of infants at high familial likelihood for autism spectrum disorder (ASD) suggest that connectivity differences among social-emotional, sensory-processing, and higher-order regulatory systems may already be present early in postnatal life, although these differences show clear age and network specificity. A study of term-born neonates found weaker connectivity between the left anterior insula and left amygdala in the high-familial-likelihood group, with weaker connectivity associated with higher social-communication risk scores at approximately 17 months of age. However, the study had a small sample, and its findings therefore provide primarily preliminary evidence of a prospective association (Scheinost et al., 2022b). In 6-week-old infants, Tsang et al. (2024) further observed a relative trade-off pattern characterized by stronger connectivity between the salience network and sensorimotor regions but weaker connectivity with prefrontal regions. Within the high-likelihood group, some connectivity features were also associated with sensory processing at 6 months and social-behavioural measures at 12 months, suggesting that the early configuration of salience-network connectivity with sensory-related and higher-order social-emotional systems may be related to subsequent differences in sensory responses and social behaviour. In contrast, 12-month-old infants at high genetic likelihood showed weaker right amygdala-left visual cortex and left amygdala-right anterior cingulate cortex connectivity. Of these findings, the association between amygdala-visual cortex connectivity and lower concurrent motor and communication scores was relatively robust (Liu et al., 2024).

Thalamocortical connectivity is another repeatedly examined early feature. In 52 infants aged 6 weeks, Nair et al. (2021) found weaker thalamic-prefrontal connectivity but stronger thalamic-occipital and thalamic-motor cortex connectivity in the high-familial-likelihood group, with some connectivity measures associated with atypical social development between 9 and 36 months of age. Wagner et al. (2023) found stronger thalamic-limbic connectivity in high-likelihood infants at 1.5 months, whereas the 9-month sample was characterized primarily by weaker thalamic-prefrontal and thalamic-motor connectivity. Their brain-behaviour analyses further showed that lower sensory over-responsivity at 6 months was associated with stronger thalamic connectivity with higher-order prefrontal regions, whereas greater sensory over-responsivity was associated with stronger thalamic connectivity with primary sensory and subcortical regions. Taken together, some thalamic studies point to a relative pattern of stronger coupling with primary sensory or subcortical systems and weaker coupling with higher-order prefrontal systems. This pattern may be related to sensory over-responsivity and later ASD-related behavioural scores, but requires further longitudinal validation. Rolison et al. (2022) found no significant group differences in the examined regions at 1 month of age. By 9 months, however, hemispheric asymmetry in the posterior cingulate and extrastriate visual cortices differed in infants who were later diagnosed with ASD or had a high familial likelihood of ASD. In addition, Chen et al. (2026) studied 62 children with both fetal rs-fMRI data and parent-reported Brief Infant-Toddler Social and Emotional Assessment (BITSEA) data at 3 years of age. They found that cingulate-left temporal and right prefrontal-left opercular network pairs were associated with higher autism-related traits; however, the outcome was a parent-reported continuous trait measure rather than a clinical ASD diagnosis.

Overall, the existing evidence supports early differences in the salience network, amygdala, thalamocortical system, and hemispheric organization in infants at high familial likelihood for ASD. Their subsequent or concurrent behavioural associations are concentrated mainly in sensory over-responsivity, social communication and social visual attention, ASD-related scale scores, and motor and communication abilities. However, stable one-to-one correspondences between individual connections and behavioural outcomes have not been established. Moreover, high familial likelihood does not necessarily lead to a later ASD diagnosis, and most studies have small samples and lack validation in independent cohorts. These findings should therefore currently be regarded as candidate network features associated with early risk.

### 5.2 Candidate prognostic indicators after neonatal brain injury

In hypoxic-ischaemic encephalopathy (HIE) and other forms of acute neonatal brain injury, rs-fMRI has primarily provided network-level evidence related to injury severity. Li et al. (2019) studied 14 neonates with mild HIE and 14 with severe HIE and found that both groups retained small-world organization. However, the severe group showed lower local efficiency and clustering coefficients, fewer hub nodes, and lower nodal efficiency in parts of the temporal, opercular, and supramarginal regions. Another study, which included only 11 neonates with mild HIE and 13 with moderate-to-severe HIE, also reported between-group connectivity differences in sensorimotor, visual-limbic, and deep-grey-matter circuits, although the results were based on uncorrected statistical thresholds (Y. Wang et al., 2023). These studies support the presence of HIE-related network differences, but both lacked healthy control groups and consisted mainly of cross-sectional comparisons of injury severity.

Longitudinal studies provide more direct, although still exploratory, prognostic evidence. In a retrospective cohort of 40 neonates with acute brain injury, abnormalities in different resting-state networks showed partly domain-specific associations with neurological status at discharge, developmental delay during follow-up, and abnormalities of motor tone (Boerwinkle et al., 2022). Subsequent 2-year follow-up further showed that abnormalities in the basal ganglia, default mode, and language/frontoparietal networks had overlapping associations with mortality and cognitive or motor outcomes, whereas the resting-state seizure-onset-zone network (RS-SOZ) was associated with subsequent epilepsy (Boerwinkle et al., 2024). These results suggest that network-level abnormalities may provide prognostic information beyond conventional descriptions of structural injury. In very preterm infants, Cyr et al. (2022) found weaker bilateral motor and visual cortical connectivity in infants with high-grade brain injury. Within this injury subgroup, stronger bilateral motor-cortex connectivity at term-equivalent age was associated with better gross-motor outcomes at 2 years, whereas this relationship was not observed in preterm infants without high-grade brain injury.

### 5.3 Prospective associations with language, cognitive, and social-emotional outcomes

Early functional connectivity has also been associated with subsequent language and cognitive abilities. In a study that followed children from infancy to school age, 76 infants underwent rs-fMRI; 61 had subsequent phonological-skills data, and 41 completed a reading assessment. Within-module connectivity of the inferior frontal module in the infant language network was associated with phonological skills in kindergarten and word-reading performance at school age. Phonological skills showed a statistical mediation effect between early connectivity and subsequent reading (Tang et al., 2024). In a larger infant cohort, Wagner et al. (2026) observed synchronization and differentiation involving some short- and long-range connections within the language network during the first year. Infants grouped according to their language-development trajectories differed in cerebellar-prefrontal, inferior frontal-parietal, and middle temporal-cerebellar connectivity, and the relevant connections were associated with measures of expressive language, vocabulary, or communication at approximately 2 years of age. Fetal studies have also provided preliminary evidence. In 25 fetuses scanned between 27 and 35 gestational weeks, prenatal connectivity of the insula with the caudate nucleus and rolandic operculum was associated with language scores between 12 and 38 months of age (Cara et al., 2025).

In the cognitive and motor domains, a study of 102 very preterm infants without major focal brain injury showed that thalamic-premotor association cortex connectivity at term-equivalent age was associated with motor scores at 20 months, whereas thalamic-primary sensorimotor cortex connectivity was associated with cognitive scores (Toulmin et al., 2021). Peyton et al. (2020) also found in 62 preterm infants that connectivity of the basal ganglia with parietal, frontotemporal, or visual regions at term-equivalent age differed between infants with different general-movement performance or cognitive and motor outcomes at 2 years. Hippocampal connectivity may carry information related to memory over a longer period. A study involving 202 children and 307 scans obtained during infancy found that hippocampal connectivity features and connectivity changes during the neonatal period and first year were associated with working-memory performance at 4 years of age. However, the follow-up samples included in the two working-memory measures comprised 111 and 97 children, respectively (Liu et al., 2021). These studies show prospective associations between early connectivity and subsequent function, but most did not assess individual-level predictive performance in independent samples.

Social-emotional outcomes involve the default mode, cingulo-opercular, salience, and amygdala-related circuits. Stronger within-network connectivity of the default mode network during the neonatal period was associated with less crying or negative affect between 1 and 6 months of age (Ravi et al., 2023). In a longitudinal study of 283 children, stronger neonatal connectivity between the cingulo-opercular network and medial prefrontal cortex was associated with higher callous-unemotional traits at 3 years and with lower empathy and prosocial behaviour between 1 and 3 years; these associations remained after controlling for the corresponding parental traits and children's externalizing symptoms (Brady et al., 2024a). A smaller study found that stronger within-amygdala-network and amygdala-salience-network connectivity at 3 months was associated with less recovery of social engagement following a stressor at 6 months, although this association was no longer significant at 9 months (Hu et al., 2024). In 129 very preterm infants, specific connections of the amygdala with the parahippocampal gyrus, occipital lobe, or thalamus at term-equivalent age were associated with a composite measure of Emotion Moderation at 4–7 years of age (Kanel et al., 2022). In addition, neonatal cerebellar functional gradients predicted externalizing-behaviour scores in toddlerhood using within-sample cross-validation, but have not yet been tested in an external cohort (Kim et al., 2024). Using densely age-sampled data from 180 infants and 396 scans, Filippi et al.(2025) found that dorsal attention network-frontoparietal network (DAN-FPN) and default mode network-salience network (DMN-SN) connectivity decreased with age. A smaller reduction in DAN-FPN connectivity, together with higher initial DAN-FPN and lower initial DAN-SN connectivity, was associated with greater fearful temperament at 2 years. These findings indicate that early social-emotional outcomes are not determined by a single “emotion network”; the behavioural significance of individual connections may vary with age, measurement instrument, and study population.

### 5.4 From characterizing individual differences to candidate clinical indicators

Using the first and second segments of the same scan from 40 neonates, Q. Wang et al. (2021) were able to identify individuals from their functional connectivity, although the short interval between segments may have overestimated the stability of the connectivity features. Hu et al. (2022) subsequently analysed 806 longitudinal scans from 104 infants and found that functional connectivity after feature selection achieved an individual-identification rate of approximately 78% and retained some stability over periods of several months. In 112 infants and toddlers aged 8–26 months, Kardan et al. (2022) further achieved individual identification and age prediction. For infants not included in model training, the mean age-prediction error was approximately 3.6 months, with a coefficient of determination of 0.51. A study of 611 infants from the developing Human Connectome Project (dHCP) also showed that both structural and functional connectomes could predict postmenstrual age within a single large dataset. Deviations between connectome-estimated and actual age were also associated with some perinatal exposures and behavioural measures in toddlerhood (Sun et al., 2024).

Moving from population-level patterns to individual assessment also requires developmental heterogeneity to be addressed. Deviation from the population-average trajectory may reflect developmental risk, but it may also represent normal individual variation, and the same connectivity feature may have different meanings across infant subgroups (Gao, 2025). For example, Chen et al. (2021) identified two subgroups with distinct brain-behaviour relationships among 81 neonates. Overall neonatal functional connectivity was similar between the two subgroups, but associations between connectivity features and intelligence scores at 4 years differed and even showed opposite directions. Although this exploratory finding requires validation in larger samples, it indicates that individualized assessment cannot be based solely on the extent to which an individual deviates from the population mean. It is also necessary to determine whether such deviations have consistent developmental significance across different clinical and environmental contexts.

At present, several barriers still separate early-life rs-fMRI from routine clinical use. First, sleep stage, head motion, physiological fluctuations, effective scan duration, head position within the coil, brain parcellation, and preprocessing strategy can all affect individual functional connectivity estimates, and the stability of these estimates across time, devices, and centres remains insufficient (King et al., 2023; Y. Wang et al., 2021). Second, most existing studies rely on within-sample associations or internal cross-validation in a single cohort and lack multicentre external validation, model calibration, and decision thresholds with clear clinical significance. Third, prematurity, underlying disease, treatment exposure, and social-environmental factors often coexist. Together with subgroup heterogeneity in brain-behaviour relationships, this makes the aetiological specificity and individual interpretation of candidate connectivity features more difficult. Clinical translation will also require normative references adapted to gestational age, postmenstrual age, and scanning state, as well as evidence that functional connectivity provides stable incremental value beyond gestational age, clinical history, neurobehavioural assessment, and structural MRI. Accordingly, current findings from individual identification, brain-age prediction, and brain-behaviour associations are more appropriately regarded as candidate research indicators and cannot yet be used directly as clinical biomarkers for individual diagnosis or treatment decisions.

## 6 Methodological challenges and future directions

Early functional-connectivity research has developed a range of age-appropriate acquisition and analytical approaches. However, the principal bottleneck in the field is not the lack of increasingly complex connectivity metrics, but rather the need to establish that the measured signals have an interpretable physiological basis and to ensure comparability across ages, cohorts and analytical pipelines. Brain structure, haemodynamics and behavioural state change rapidly during infancy, and imaging is often acquired during natural sleep. Sleep state, head motion, respiration-related artefacts, head position within the coil, effective scan duration, preprocessing strategies and brain parcellation can therefore all influence estimates of functional connectivity.

### 6.1 Sleep and wake states

Natural sleep can reduce head motion in infants, but it should not be regarded as a neutral “resting” condition equivalent to wakefulness. Mitra et al. (2017) compared rs-fMRI data from sleeping infants aged 6, 12 and 24 months with data from adults during wakefulness and non-rapid eye movement (NREM) sleep. Across all three infant age groups, the overall functional-connectivity matrices more closely resembled those of adults during NREM sleep than those of awake adults. The relative attenuation of the default mode network in sleeping infants and sleeping adults also suggested that some differences between infants and awake adults might be related to scanning state. However, patterns of BOLD-signal propagation were region- and age-specific. For example, at 6 months, some thalamic propagation features remained closer to those observed in awake adults and only subsequently shifted towards the adult sleep pattern. These findings indicate that infant–adult connectivity differences may reflect both developmental and state-of-consciousness effects, and that these effects are not uniform across connectivity measures.

Yates et al. (2023) further compared functional connectivity in infants during natural sleep and awake viewing of films. Whole-brain connectivity patterns distinguished the two states, and some connections involving frontoparietal control networks also varied with state. However, the sleep and wake samples only partially overlapped, and the age range was broad. The study therefore should not be described as a strict within-subject comparison of sleep and wakefulness.

Sleep itself comprises multiple physiological states. Using simultaneous EEG and fNIRS, Lee et al. (2020) found stronger interhemispheric connectivity during active sleep (AS) in term neonates, whereas quiet sleep (QS) was characterised by more prominent short-range intrahemispheric connectivity. Shiraki et al. (2024) further showed, in 63 preterm and 44 term infants, that periodic breathing could inflate functional-connectivity estimates. After the corresponding segments were excluded, some connections remained stronger during AS than QS, particularly connections involving the occipital cortex. Because both studies used EEG–fNIRS, their findings demonstrate that sleep stage and respiratory pattern can influence neonatal functional coupling, but they do not provide direct equivalent evidence for rs-fMRI.

Mueller et al. (2025) attempted to classify sleep state during rs-fMRI using simultaneously recorded respiratory signals. Among 20 infants near term-equivalent postmenstrual age, 11 had respiratory recordings of sufficient quality, but only four entered QS at any point during functional scanning. The study found no AS–QS connectivity differences that remained significant after correction for multiple comparisons, although some connections showed relatively large effect sizes. These findings primarily support the feasibility of using respiratory signals to assist in classifying scanning state; they do not establish that AS and QS are not associated with differences in functional connectivity.

Insufficient recording of sleep state is a widespread limitation in this field. Carnevali et al. (2026) systematically reviewed 103 task-free functional-connectivity studies conducted during the first 100 postnatal days. Of these, 92 were conducted exclusively during sleep, seven included both sleep and wakefulness, and four included wakefulness only. However, only 18 studies further determined the specific sleep stage, and among 73 fMRI studies, only five recorded or distinguished specific sleep states. Future studies should, whenever possible, record EEG, respiration, heart rate and eye movements simultaneously, and report the duration of usable data obtained during each sleep stage and state transition. fMRI in awake infants has been shown to be feasible (Ellis et al., 2020), but remains affected by head motion, difficulty sustaining attention and selective missingness.

### 6.2 Head motion, physiological noise and preprocessing

Resting-state fMRI functional connectivity reflects statistical covariation between spontaneous blood oxygenation level-dependent (BOLD) time series rather than direct structural connections or information transfer between neurons. Because tissue contrast, haemodynamics, physiological frequencies and motion characteristics differ between infants and adults, registration, filtering and denoising parameters commonly used in adult studies should not be applied directly to infant data without validation (Cusack et al., 2018).

Kim et al. (2023) analysed 159 neonatal scans and 416 dHCP scans and found associations between head motion and connectivity within sensory networks, the default mode network and whole-brain connectivity. The direction of these effects varied across brain regions. Censoring high-motion volumes attenuated these associations, but residual motion–connectivity relationships remained detectable after standard dHCP denoising.

Fetal studies have yielded similar findings. Kim et al. (2025) analysed 120 scans from 104 fetuses and found that regression of motion parameters reduced local BOLD fluctuations and the associations between some connectivity measures and head motion. However, identifiable motion-related information remained in whole-brain connectivity patterns. Censoring high-motion volumes further reduced bias, but overly stringent thresholds substantially shortened the effective data duration and reduced the number of analysable samples.

Taymourtash et al. (2025) used data from 70 fetuses at 19–39 gestational weeks and a generative model to compare 11 commonly used regression and denoising strategies. Within the evaluation framework of that study, volume censoring, global signal regression and anatomical-compartment-based component regression were relatively effective in reducing residual motion effects. Nevertheless, the study used a single cohort and one fetal brain parcellation, and different methods may alter the distribution of connection lengths, the neural signal and sample retention. These findings support the use of multi-pipeline sensitivity analyses, but do not identify a single optimal strategy applicable to all fetal datasets.

Head position within the coil is another easily overlooked confounding factor. King et al. (2023) found that the position of an infant’s head within the coil altered the spatial distribution of the signal-to-noise ratio and significantly affected connectome-fingerprinting results obtained during the same acquisition period. Accordingly, studies of individual identification and test–retest reliability should assess head position, coil coverage and regional signal-to-noise ratio in addition to head motion.

Age-appropriate pipelines may improve processing consistency. The dHCP automated framework integrates slice-to-volume motion correction, dynamic susceptibility-distortion correction, multimodal registration, ICA-based denoising and automated quality control (Fitzgibbon et al., 2020). NeoRS provides a more readily deployable neonatal preprocessing pipeline, although its initial evaluation included only 10 neonates (Enguix et al., 2022). These pipelines do not yet constitute a universal standard applicable to all ages and acquisition protocols.

### 6.3 Brain parcellation, individualised mapping and dynamic connectivity

Brain parcellation directly affects the identification of network nodes, modules, connection distances and hubs. Myers et al. (2024) found that adult and older-infant surface parcellations, as well as neonatal volumetric parcellations, did not adequately match the functional boundaries of the neonatal cortical surface. The study subsequently established a surface atlas containing 283 cortical parcels based on 261 neonates and validated it in three external neonatal datasets. These findings support the use of age-appropriate atlases, but do not imply that a single fixed parcellation can be shared across different gestational and postnatal ages.

Individualised mapping may complement group-level atlases. Derman and Ferradal (2025) applied individualised network mapping to 369 valid scans from 352 term and preterm infants and showed that group averaging may attenuate spatial differences in higher-order association networks. However, the longitudinal subset with true repeated scans was small. Labonte et al. (2026) conducted high-density scanning across different days in eight neonates, of whom six were included in the primary analysis. Approximately 42 min of data yielded moderate-to-high within-subject connectivity repeatability, but the sample was very small and sleep stage was not objectively determined.

Dynamic functional connectivity (dFC) can complement conventional time-averaged connectivity. França et al. (2024) identified six transient connectivity states in 324 term and 66 preterm infants and found that some state features were associated with prematurity and Bayley-III and Q-CHAT scores at 18 months. At the same time, the number of dynamic states and their transition characteristics are influenced by model parameters, effective data length, head motion, physiological fluctuations and transitions between sleep states. dFC should therefore be regarded as a complementary research measure.

### 6.4 Cross-study heterogeneity, reproducibility and translational boundaries

This review spans studies of fetuses, preterm infants, term neonates and infants. Although this integration helps delineate the overall developmental trajectory of early functional networks, it also limits direct comparisons across studies. Studies may differ in sleep, wake or sedation state; scanner and coil; temporal resolution; scan duration; motion-exclusion criteria; global-signal processing; brain parcellation; connectivity metrics; and statistical thresholds.

Large or multicentre datasets can increase statistical power, but they cannot automatically eliminate methodological heterogeneity. Y. Wang et al. (2021) analysed 119 infants from four studies and found low same-scan test–retest reliability for individual connections, whereas the reliability of individual whole-connectome patterns was moderate to good. Reliability estimates also varied with data duration, research centre and scanner manufacturer. Future studies should make quality-control results, processing code and analytical parameters available, and should assess the stability of findings through cross-pipeline sensitivity analyses, multicentre harmonisation and independent external validation.

Brain–behaviour associations are also constrained by follow-up intervals, measurement instruments and sources of information. Infant rs-fMRI and caregiver reports, experimental tasks or clinical ratings obtained months or years later are not measured concurrently. Statistical associations therefore cannot establish that a particular connection directly supports or causes the corresponding behaviour. Future research should pre-specify primary outcomes and clearly distinguish within-sample associations, internal cross-validation and independent external validation. These steps would provide a more reliable evidence base for the individual interpretation and subsequent translation of early functional connectivity.

## 7 Conclusion

Resting-state fMRI studies indicate that large-scale functional brain organisation is detectable before birth and undergoes rapid reorganisation during the perinatal period and the first postnatal year. Taken together, the evidence suggests that primary sensory and motor systems tend to establish relatively stable functional organisation earlier, whereas higher-order association networks and long-range coordination across networks undergo more prolonged refinement. Prematurity, as well as prenatal, maternal, social and clinical environments, is associated with differences in early functional connectivity. Some fetal, neonatal and infant connectivity measures also show prospective associations with later cognitive, language, motor and social-emotional outcomes.

The interpretation and translation of these findings remain constrained by the predominance of cross-sectional studies, limited representativeness of study populations, and substantial heterogeneity in sleep and wake state, head-motion and physiological monitoring, acquisition protocols, preprocessing strategies, brain parcellation, connectivity metrics and outcome assessment. Future studies should prioritise repeated measurements in the same individuals, establish better-coordinated multicentre cohorts, improve population representativeness, objectively characterise sleep and physiological states, and replicate brain–behaviour associations in independent samples. Guided by clearly specified hypotheses, the integration of rs-fMRI with structural and diffusion MRI, EEG or fNIRS, physiological recordings and repeated behavioural assessments may help clarify the biological basis and incremental value of functional-connectivity measures.

## CRediT authorship contribution statement

**Chang Liu:** Writing – review & editing. **Rong Ju:** Writing – review & editing, Supervision, Conceptualization. **Fangwei Yuan:** Visualization, Investigation. **Xinyu Lin:** Data curation. **Xin Tang:** Writing – review & editing, Writing – original draft, Investigation.

## Declaration of Generative AI and AI-assisted technologies in the writing process

During the preparation of this work, the authors used ChatGPT to improve language clarity and readability. After using this tool, the authors reviewed and edited the content as needed and took full responsibility for the content of the published article.

## Funding

This work was supported by the following projects from Chengdu Women's and Children's Central Hospital: the Clinical Research Project on Non-invasive CVP in Neonates (Grant No. 2023010), the project entitled “Effects of Carbon Dioxide Partial Pressure and Blood Pressure on Cerebral Blood Flow in Extremely Preterm and Very Preterm Infants” (Grant No. 2023001), and the YingCai Cultivate Program (Grant No. YC2022004).

## Declaration of Competing Interest

The authors declare that they have no known competing financial interests or personal relationships that could have appeared to influence the work reported in this paper.

## Data Availability

No data was used for the research described in the article.
